# Predicting the risk of mortality during hospitalization in sick severely malnourished children using daily evaluation of key clinical warning signs

**DOI:** 10.1186/s12916-021-02074-6

**Published:** 2021-09-20

**Authors:** Bijun Wen, Daniella Brals, Celine Bourdon, Lauren Erdman, Moses Ngari, Emmanuel Chimwezi, Isabel Potani, Johnstone Thitiri, Laura Mwalekwa, James A. Berkley, Robert H. J. Bandsma, Wieger Voskuijl

**Affiliations:** 1 Department of Nutritional Sciences, Faculty of Medicine, University of Toronto, Toronto, Canada; 2 Division of Gastroenterology, Hepatology and Nutrition, The Hospital for Sick Children, Toronto, Canada; 3 Amsterdam Institute for Global Health and Development, Department of Global Health, Amsterdam University Medical Centres, Amsterdam, The Netherlands; 4 The Childhood Acute Illness & Nutrition Network, Nairobi, Kenya; 5 Genetics and Genome Biology Program, The Hospital for Sick Children, Toronto, Canada; 6 Clinical Research Department, KEMRI/Wellcome Trust Research Programme, Kilifi, Kenya; 7 Department of Paediatrics, Kamuzu University of Health Sciences, formerly College of Medicine, University of Malawi, Blantyre, Malawi; 8 Department of Paediatrics, Coast General Hospital, Mombasa, Kenya; 9 Centre for Tropical Medicine & Global Health, Nuffield Department of Medicine, University of Oxford, Oxford, UK; 10 Department of Biomedical Sciences, Kamuzu University of Health Sciences, formerly College of Medicine, University of Malawi, Blantyre, Malawi; 11 Amsterdam Center for Global Child Health, Emma Children’s Hospital, Amsterdam University Medical Centres, Meibergdreef 9, 1105 AZ Amsterdam, The Netherlands

**Keywords:** Severe malnutrition, SAM, Mortality prediction, Danger signs, Sub-Saharan Africa

## Abstract

**Background:**

Despite adherence to WHO guidelines, inpatient mortality among sick children admitted to hospital with complicated severe acute malnutrition (SAM) remains unacceptably high. Several studies have examined risk factors present at admission for mortality. However, risks may evolve during admission with medical and nutritional treatment or deterioration. Currently, no specific guidance exists for assessing daily treatment response. This study aimed to determine the prognostic value of monitoring clinical signs on a daily basis for assessing mortality risk during hospitalization in children with SAM.

**Methods:**

This is a secondary analysis of data from a randomized trial (NCT02246296) among 843 hospitalized children with SAM. Daily clinical signs were prospectively collected during ward rounds. Multivariable extended Cox regression using backward feature selection was performed to identify daily clinical warning signs (CWS) associated with time to death within the first 21 days of hospitalization. Predictive models were subsequently developed, and their prognostic performance evaluated using Harrell’s concordance index (C-index) and time-dependent area under the curve (tAUC).

**Results:**

Inpatient case fatality ratio was 16.3% (*n*=127). The presence of the following CWS during daily assessment were found to be independent predictors of inpatient mortality: symptomatic hypoglycemia, reduced consciousness, chest indrawing, not able to complete feeds, nutritional edema, diarrhea, and fever. Daily risk scores computed using these 7 CWS together with MUAC<10.5cm at admission as additional CWS predict survival outcome of children with SAM with a C-index of 0.81 (95% CI 0.77–0.86). Moreover, counting signs among the top 5 CWS (reduced consciousness, symptomatic hypoglycemia, chest indrawing, not able to complete foods, and MUAC<10.5cm) provided a simpler tool with similar prognostic performance (C-index of 0.79; 95% CI 0.74–0.84). Having 1 or 2 of these CWS on any day during hospitalization was associated with a 3 or 11-fold increased mortality risk compared with no signs, respectively.

**Conclusions:**

This study provides evidence for structured monitoring of daily CWS as recommended clinical practice as it improves prediction of inpatient mortality among sick children with complicated SAM. We propose a simple counting-tool to guide healthcare workers to assess treatment response for these children.

**Trial registration:**

NCT02246296

**Supplementary Information:**

The online version contains supplementary material available at 10.1186/s12916-021-02074-6.

## Background

Undernutrition accounts for 45% of deaths in children under 5 years of age globally [1]. Severe acute malnutrition (SAM) is the most life-threatening form of undernutrition and is defined by the World Health Organization (WHO) as either a weight-for-height *Z* score (WHZ) < -3 or a mid-upper arm circumference (MUAC) <11.5 cm, or the presence of bilateral pitting edema [2]. SAM is a multi-factorial condition arising from the interplay between food insecurity, poverty, and acute or chronic disease, and despite its name, often does not occur acutely [3]. Children with complicated SAM (i.e., with medical complications, usually serious infections) require hospital admission to manage life-threatening conditions in addition to nutritional rehabilitation. The WHO has standardized management guidelines for children with complicated SAM [4]. However, inpatient case-fatality rates for children with SAM remain unacceptably high at 10–25% in African. Apart from factors inherent to low-resource settings, the high mortality is related to poor understanding of the pathophysiology and weakly evidence-based treatment protocol [5–7].

A number of risk factors have been associated with mortality in SAM, including infections (e.g., pneumonia, malaria, and HIV), edema, and metabolic disturbances (e.g., hypoglycemia and possibly refeeding syndrome) [3]. In addition, studies have reported that more than 50% of hospitalized children with SAM have diarrhea, which may lead to dehydration and shock that are difficult to manage, increasing the risk of mortality [8, 9].

Obstructed breathing, severe respiratory distress, severe anemia, shock, reduced consciousness, seizures, diarrhea, and signs of severe dehydration are defined by the WHO as “clinical danger signs” that are presumptively predictive for clinical deterioration and mortality [10, 11]. These clinical danger signs are indicators of disease severity and used in emergency triage assessment and treatment (ETAT) for all hospitalized children in low-resource settings, whether or not they are malnourished. Although considered useful in identifying children requiring immediate hospital care, only a few studies have specifically evaluated their prognostic values in children with SAM [12–15], who have the highest risk of dying. Importantly, studies conventionally report on the use of these clinical signs for prognosis upon hospital admission only. However, mortality risk is expected to evolve during hospitalization for SAM, as children may improve or deteriorate during admission despite strict adherence to protocolized medical and nutritional treatment [3]. Although clinicians typically assess their patients’ status by monitoring daily clinical signs, no evidence based, structured guidance exists for such assessment, and no study has evaluated the value of structured monitoring of clinical signs every day during hospitalization.

To address this important research gap, we first determined a set of daily clinical warning signs (CWS) most predictive for inpatient mortality of children with SAM using data collected during daily ward rounds during multi-center study clinical trial. Secondly, we evaluated the prognostic value of the identified CWS as an easily applicable tool to indicate patient’s risk for mortality during hospitalization.

## Methods

### Study design and participants

This was a secondary analysis of data collected during a randomized double-blinded clinical trial (NCT02246296) among 843 SAM patients in two hospitals in Kenya and one in Malawi [16]. The trial was designed to determine if a modified carbohydrate reduced F75 rehabilitation milk formula would decrease the time to clinical and nutritional stabilization compared to the current standard F75 formula. Inclusion criteria were age 6–156 months, classified as SAM [i.e., either MUAC<11.5cm (for age<60months) or WHZ< -3 (for age<60months) or BMI-for-age *Z* score< -3 (for age≥60 months) or bilateral pitting edema] with either medical complications (complicated SAM; with medical complications like systemic or respiratory infection, gastroenteritis, or HIV disease) or failing an appetite test as per WHO guidelines [17]. All patients received standard care in accordance with the WHO and national guidelines. Informed consent was obtained from parents or caregivers prior to enrollment in the trial. Ethical approval was obtained from the College of Medicine Research and Ethics Committee of the University of Malawi, the KEMRI Ethical Review Committee, Kenya, the Oxford Tropical Research Ethics Committee, and the Hospital for Sick Children, Toronto, Canada.

### Data collection and study variables

Data were recorded and maintained in compliance with ICH E6 GCP as well as regulatory and institutional requirements for the protection of patient confidentiality. Upon admission, we collected patients’ demographics, and both on admission and during daily ward rounds, we collected 11 clinical signs (assessing if these clinical signs were present during the last 24 h) using a standard proforma (Additional file 1: Table S1): reduced consciousness (P or U on the AVPU-scale [11], lower chest wall indrawing, shock (fast and weak pulse, cold hands, and capillary refill time >3 s), convulsions, vomiting, diarrhea (>3 loose/watery stools), hypothermia (temperature <36.5^o^C), fever (temperature >38.5^o^C), symptomatic hypoglycemia (<3 mmol/l, glucose was measured systematically at admission then only when clinicians suspected hypoglycemia), nutritional edema, and not being able to complete feeds. All clinical assessments were performed by medical study staff who received training to standardize recognition of clinical signs and recording across the sites. On the case report form all clinical signs were recorded as discrete categorical variables (present versus not present). Not able to complete feeds was assessed by trained study staff during a feeding observation. Intake was subsequently scored as 25%, 50%, 75%, and 100% of the milk/RUTF. Such feeding observations were done if the attending clinician doubted if a child was finishing the prescribed amount of milk/RUTF. Intake of 75% or less of the prescribed amount of milk/RUTF was considered as not able to complete feeds. If children consumed 50% or less, they were given an NG tube. HIV testing by rapid antibody test was offered to all participants according to national guidelines, with appropriate counseling, follow-up tests, and referrals offered depending on results. Malaria was diagnosed in all children using blood smears, or when a slide could not be immediately done a rapid diagnostic test was performed.

### Identification of clinical warning signs predictive for mortality

All analyses were performed using R statistical software (version 3.4.3; R Development Core Team, 2017) [18]. Descriptive statistics were used to summarize baseline characteristics of the study population. Mean and standard deviation (SD) were calculated for continuous variables, and the number of patients (*n*) and percentages (%) was presented for categorical variables. The outcome of interest was defined as the number of days between admission and inpatient death (time-to-event), and hospital discharge was right censored. As the last death occurred on hospitalization day 20, we did not include data after day 21. Because of their clinical relevance for mortality prediction, MUAC (as a continuous variable) at admission and HIV status (HIV−, HIV+/exposed, or refused testing/died before testing) at admission were considered as a priori time-constant predictors in all explanatory models, irrespective of whether they were statistically significant [19]. MUAC was chosen as this measure is less affected by dehydration than weight-based anthropometry [20].

To evaluate the average effects of the time-varying clinical signs on the outcome, explanatory survival analysis was performed to estimate the daily cause-specific hazards ratios for mortality (HR_death_) with corresponding 95% confidence intervals (95% CIs). In order to take into account the time-varying nature of the 11 clinical signs, a multivariable extended Cox proportional hazards (PH) model was employed [21, 22]. This explanatory model included the a priori predictors, the 11 time-varying clinical signs and was further adjusted for other time-invariant potential predictors including sex, age, study site, treatment arm of the trial and known comorbidities [cerebral palsy, severe pneumonia, severe anemia (Hb <5g/dl), and malaria] (Full Mortality Model). We used the 2013 modified WHO definition of severe pneumonia requiring hospital admission due to the presence of cough or difficulty in breathing and tachypnoea. In addition, the clinically plausible interactions between age and HIV status and between age and MUAC were tested in this model.

To determine the subset of daily CWS predictive for mortality, a backward feature selection procedure based on Akaike information criterion was performed on the Full Mortality Model [23]. The identified CWS were subsequently fitted to a multivariable extended PH model, where features violating the PH assumption were included with time-dependent coefficients, modeled as a linear function of admission duration (Reduced Mortality Model).

We acknowledged that hospital discharge could be a competing risk event precluding the occurrence of inpatient mortality. However, employing the Fine-Gray sub-distribution hazard model to time-varying covariates demands extreme caution, with the loss of ability to estimate the cumulative incidence function, as previously described by Austin et al. [24] and Poguntke et al. [25]. Therefore, to examine the influence of competing risk effect from hospital discharge, we performed the following sensitivity analyses. First, cause-specific hazard ratios for hospital discharge (HR_discharge_) were estimated using a multivariable extended Cox PH model, treating hospital discharge as event of interest and inpatient mortality as censored (Reduced Discharge Model). Second, after manually setting the unobserved clinical signs after discharge to two extreme opposites, the Reduced Mortality Model was re-estimated twice. First where we assumed no signs were present after discharge (Reduced Mortality Model: scenario 1) and second assuming the signs present at discharge carried on until day 21 (Reduced Mortality Model: scenario 2). This allowed us to investigate the limitation of not observing clinical signs after discharge but imposes another strong assumption that post-discharge mortality did not occur in our population.

For model diagnostics, potential multicollinearity between predictors was accessed by the variance inflation factor (VIF) [23]. Influential observations were detected based on the difference in the β coefficient (DFBETA) statistics with threshold at 0.4. The PH assumption was checked by the Scaled Schoenfeld residuals test. Additional sensitivity analyses were conducted to test robustness of results when the influential observations were removed, or when discrete-time models were used in the survival analysis [26].

### Development and evaluation of predictive models

To examine the value of using the identified CWS for assessing patient status daily during hospitalization, we developed and compared four predictive models for mortality. The a priori and other potential predictors measured only at admission were included in the predictive models if selected by the Reduced Mortality Model, which resulted in MUAC at admission being chosen. For the purpose of developing an easily applicable prediction tool, the continuous variable MUAC at admission was dichotomized into a categorical variable—very severe wasting (MUAC<10.5) or not (MUAC ≥ 10.5), when building the following predictive models. First, we evaluated how well admission data alone can predict ultimate survival outcome, imitating what is commonly evaluated in the current literature. To this end, we built a predictive model with the admission score of the identified daily CWS together with MUAC<10.5cm (Predictive Model 1: Admission Score), using multivariable Cox PH regressions [27]. Second, we evaluated how well the daily data discriminated mortality risk, by building a predictive model using the identified daily CWS plus MUAC<10.5cm at admission as an additional time-invariant CWS, using multivariable extended Cox PH regression (Predictive Model 2: Daily Score) [21, 22]. Using this model, the time-updated risk scores that reflect each patient’s daily instantaneous hazard of dying were estimated based on model estimates *Xβ* (i.e., model-based scores). Third, we investigated the potential of using the daily count of the number of CWS (i.e., 0, 1, 2, 3, and >3) as a simplified tool for mortality risk prediction, where the counted number of daily CWS was increased by 1 on each hospitalization day if the child had a MUAC<10.5cm at admission (Predictive Model 3: Daily Count). Lastly, we evaluated the potential of further reducing the list of CWS to 5 as the simplest tool for mortality risk prediction (Predictive Model 4: Daily Top 5 Count). With this approach, the top 5 CWS were counted among the identified daily CWS and MUAC<10.5cm at admission as additional time-invariant CWS, and their order of importance was determined in Predictive Model 2 based on decreasing HR. Associations between CWS counts and mortality were assessed by extended Cox PH regressions [21, 22]. It is worth noting that these counting tool models represent a more applicable approach for patient assessment compared to Predictive Model 2, since risk scores equal directly to the total number of the CWS observed while complex mathematical computation (i.e., model-based) is not needed.

To compare the discriminant performance of risk scores predicted by each model, we evaluated Harrell’s concordance index (C-index) using the “rms” R package [23]. Bootstrap validation was conducted with 1000 repetitions to examine the internal validity of the models, correct for optimism, as well as estimate 95% CIs for the C-index. Additional file 2: Figure S1 illustrates how the C-index is computed for Cox PH and extended Cox PH models. Briefly, the C-index of time-static Cox PH model (e.g., Predictive Model 1: Admission Score) evaluates the performance of using risk scores at a specific time point to predict survival outcome by the end of the study follow-up, whereas the C-index of the time-updated extended Cox PH model (e.g., Predictive Model 2: Daily Score) evaluates the average performance of using the daily risk scores to predict survival outcome by the end of that respective day [28]. As a sensitivity analysis, we compared the C-index of Predictive Model 4 derived from models including our study population (aged 6–156 months) to that derived from children aged 6 to 59 months (the typical age range of SAM).

Although being able to capture the instantaneous risk of dying on each day is an important feature for a daily assessment tool, it is also clinically relevant to know the performance of risk scores for mortality occurring several days after the score day. To further assess the prognostic performance of risk scores on a specific day as the length of prediction time window increases, the time-dependent area under the receiver operating characteristic curve (t-AUC) was assessed using the “timeROC” R package as described by Blanche et al. [29].

## Results

### Admission characteristics

With 63 (7.5%) patients who withdrew from the trial, data of the remaining 780 (92.5%) patients were analyzed, including 290 (37.2%) from Coast Provincial General Hospital, 179 (22.9%) from Kilifi County Hospital, and 311 (39.9%) from Queen Elizabeth Central Hospital (Table 1). All study participants were SAM per the WHO criteria (i.e., based on MUAC, WHZ, or edema) with 12%, 16%, and 17% of participants classified as SAM by MUAC alone, WHZ alone, and edema alone, respectively, 40% by both MUAC and WHZ, and the remaining 15% by edema and MUAC, or edema and WHZ, or all three together. The large overlap of children meeting the criteria of both MUAC and WHZ indicates a highly vulnerable study population. The median age of the patients was 16.9 months (IQR 10.8–26.5) and 420 (53.8%) were males. The median MUAC of non-edematous children was 11.0 (IQR 10.4–11.5). At hospital admission, 169 children (21.7%) had a positive HIV antibody test (HIV+/exposed) and 40 (5.1%) had a declined or missed HIV test (HIV refused/died before testing). Cerebral palsy, severe pneumonia, severe anemia, and malaria were observed in 116 (14.9%), 193 (25%), 26 (3.3%), and 63 children (8.1%), respectively.

**Table 1:** Baseline patient characteristics (upon admission)

	Total SAM patients	Discharged	Died	*P*
	(*n*=780)	(*n*=653)	(*n*=127)	
***Study related characteristics***				
Hospital stay (days), median (IQR)	8.0 (6.0, 11.0)	8 (6.0, 12.0)	5 (3.0, 9.0)	<0.001
Study site, n (%)				
Coast Provincial General Hospital	290 (37.2)	250 (38.3)	40 (31.5)	
Kilifi County Hospital	179 (22.9)	156 (23.9)	23 (18.1)	
Queen Elizabeth Central Hospital	311 (39.9)	247 (37.8)	64 (50.4)	0.03
Treatment arm of trial, n (%)	390 (50.0)	322 (49.3)	68 (53.5)	0.38
*Demographic and anthropometric characteristics*				
Age in months, median (IQR)	16.9 (10.8, 26.5)	17.1 (11.0, 26.3)	15.7 (10.1, 26.8)	0.45
Age 6-59 months, n (%)	738 (94.6)	615 (94.2)	123 (96.9)	0.22
Male, n (%)	420 (53.8)	357 (54.7)	63 (49.6)	0.3
MUAC in cm (non-edematous), median (IQR)	*n*=534; 11.0 (10.4, 11.5)	*n*=454; 11.0 (10.5, 11.6)	*n*=80; 10.5 (9.5, 11.0)	<0.001
MUAC in cm, median (IQR)	11.2 (10.5, 12.0)	11.2 (10.5, 12.0)	10.8 (9.8, 11.4)	<0.001
MUAC<10.5cm, n (%)	192 (24.6)	141 (21.6)	51 (40.2)	<0.001
HAZ, median (IQR)	*n*=776; -3.0 (-4.3, -1.9)	*n*=124; -3.4 (-4.6, -2.0)	*n*=652; -2.9 (-4.2, -1.8)	0.067
WAZ, median (IQR)	*n*=778; -3.9 (-4.9, -3.1)	*n*=651; -3.9 (-4.8, -3.0)	-4.5 (-5.4, -3.6)	<0.001
WHZ, median (IQR)	*n*=737; -3.5 (-4.2, -2.7)	*n*=617; -3.4 (-4.1, -2.6)	*n*=120; -3.9 (-4.7, -3.0)	<0.001
*Comorbidities (observed upon admission only)*				
HIV status, *n* (%)				
HIV-	571 (73.2)	507 (77.6)	64 (50.4)	
HIV+/exposed	169 (21.7)	122 (18.7)	47 (37.0)	
Refused testing/died before testing	40 (5.1)	24 (3.7)	16 (12.6)	<0.001
Cerebral palsy, n (%)	116 (14.9)	99 (15.2)	17 (13.4)	0.61
Severe pneumonia, n (%)	193 (24.7)	153 (23.4)	40 (31.5)	0.054
Severe anemia, n (%)	26 (3.3)	22 (3.4)	4 (3.1)	0.9
Malaria, n (%)	63 (8.1)	57 (8.7)	6 (4.7)	0.13
***Prevalence at admission of clinical signs observed daily***				
Chest indrawing, n (%)	144 (18.5)	105 (16.1)	39 (30.7)	<0.001
Convulsions, n (%)	37 (4.7)	30 (4.6)	7 (5.5)	0.66
Diarrhea, n (%)	328 (42.1)	267 (40.9)	61 (48.0)	0.14
Fever, n (%)	216 (27.7)	180 (27.6)	36 (28.3)	0.86
Symptomatic hypoglycemia, n (%)	13 (1.7)	6 (0.9)	7 (5.5)	<0.001
Hypothermia, n (%)	43 (5.5)	35 (5.4)	8 (6.3)	0.67
Nutritional edema, n/total non-missing (%)	246/777 (31.6)	47 (37.0)	199/650 (30.6)	0.13
Not able to complete feeds, n/total non-missing (%)	450/773 (58.2)	74/126 (58.7)	376/647 (58.1)	0.9
Reduced consciousness, n (%)	24 (3.1)	13 (2.0)	11 (8.7)	<0.001
Shock, n (%)	25 (3.2)	16 (2.5)	9 (7.1)	0.007
Vomiting, n (%)	215 (27.6)	183 (28.0)	32 (25.2)	0.52

Notes: data are median (IQR) or number (%) of SAM patients, shown for all patients as well as by outcome (discharged vs. died). *HAZ* Height-for-age *Z* score. *WAZ* Weight-for-age *Z* score. *WHZ* Weight-for-height *Z* score. *HIV-* HIV-negative. *HIV+/exposed* HIV-positive or positive antibody reactivity

### Survival outcome

The median length of hospitalization was 8 days (IQR 6~12). During the study, 127 (16.3%) children died and 653 (83.7%) recovered and were discharged. The mean survival time for the children who died was 7 days (including admission day), where 14.2% of inpatient deaths took place in the first 48 h, 61% within the first 7 days, and 90% within 14 days of admission. The Kaplan-Meier estimate of event-free probability was 0.9 (95% CI 0.88–0.93) on the 5th day of admission and 0.8 (95% CI 0.76–0.84) on the 11th day for all patients (before restricting data to 21 days), assuming no post-discharge deaths. The median survival time for the population is indeterminate because the survival probability remained above 50% by the end of study (Additional file 3: Figure S2).

### Prevalence of clinical signs

At admission, the observed clinical signs included not able to complete foods (58.2%), diarrhea (42.1%), fever (27.7%), nutritional edema (31.6%), vomiting (27.6%), chest indrawing (18.5%), hypothermia (5.5%), convulsions (4.7%), shock (3.2%), reduced consciousness (3.1%), and hypoglycemia (1.7%) (Table 1).

The 780 patients in our study had the potential to generate 7025 daily data points, of which 6852 were analyzed in our models (see Additional file 4: Table S2 for percentage of missingness by CWS). The dynamics of each clinical signs during hospitalization can be found in Additional file 5: Figure S3.

### Daily CWS predictive for mortality

The a priori predictor MUAC was significantly associated with mortality (Table 2: Full Mortality Model). However, being tested HIV+/exposed at admission was not associated with mortality. Stepwise backward feature selection identified seven CWS along with (continuous) MUAC and site as the most important subset of predictors for inpatient mortality (Table 2: Reduced Mortality Model). The 7 identified daily CWS included, in order of decreasing daily HR_death_, symptomatic hypoglycemia, reduced consciousness, lower chest wall indrawing, not being able to complete feeds, nutritional edema, diarrhoea, and fever. One CWS, reduced consciousness, was associated with mortality in a time-dependent manner (*P*_*PH violation*_=0.03). The HR_death_ for reduced consciousness was 3.9 (95% CI 1.9-8.2; *P*<0.001) at the first onset and significantly increased further by 15% for each subsequent hospitalization day (Additional file 6: Figure S4).

**Table 2:** Survival analysis estimation results: adjusted effects of daily clinical signs on inpatient mortality

	Main analysis				Sensitivity analysis					
	*Full Mortality Model*		*Reduced Mortality Model*		*Reduced Discharge Model*		*Reduced Mortality Model: scenario 1*		*Reduced Mortality Model: scenario 2*	
	*Extended Cox PH with daily clinical signsEvent=death; Censored=discharge*		*Extended Cox PH with indentified daily CWSEvent=death; Censored=discharge*		*Extended Cox PH with identified daily CWSEvent=discharge; Censored=death*		*Extended Cox PH with identified daily CWSSigns set to “not present” after dischargeEvent=death; Censored=discharge*		*Extended Cox PH with selected daily CWSSigns set to discharge value (not present/present) after dischargeEvent=death; Censored=discharge*	
	HR (95% CI)	* P*	HR (95% CI)	* P*	HR (95% CI)	* P*	HR (95% CI)	* P*	HR (95% CI)	* P*
*A priori predictors (measured only at admission):*										
MUAC	0.79 (0.68–0.93)	0.01	0.80 (0.68-0.93)	0.004	1.16 (1.09–1.22)	<0.001	0.77 (0.65–0.92)	0.003	0.78 (0.66–0.92)	0.00
HIV status:										
HIV-	1.00 (ref)		1.00 (ref)		1.00 (ref)		1.00 (ref)		1.00 (ref)	
HIV+/exposed	1.36 (0.86–2.16)	0.19	1.29 (0.83–2.01)	0.26	0.85 (0.69–1.04)	0.11	1.53 (0.99–2.37)	0.06	1.55 (1–2.41)	0.05
Refused testing/died before testing	7.74 (4.17–14.38)	<0.001	7.58 (4.18–13.74)	<0.001	1.24 (0.82–1.89)	0.31	5.84 (3.64–9.35)	<0.001	5.83 (3.63–9.36)	<0.001
*Potential predictors (measured only at admission):*										
Study site:										
Coast Provincial General Hospital	1.00 (ref)		1.00 (ref)		1.00 (ref)		1.00 (ref)		1.00 (ref)	
Kilifi County Hospital	1.22 (0.68–2.20)	0.50	1.09 (0.60–1.97)	0.77	0.94 (0.76–1.17)	0.59	1.21 (0.71–2.06)	0.48	1.14 (0.67–1.95)	0.63
Queen Elizabeth Central Hospital	3.09 (1.87–5.10)	<0.001	2.90 (1.81–4.63)	<0.001	2.59 (2.14–3.13)	<0.001	2.38 (1.53–3.71)	<0.001	2.39 (1.52–3.74)	<0.001
Trial arm	1.07 (0.73–1.58)	0.72								
Age	1.01 (0.99–1.02)	0.40								
Male	1.19 (0.81–1.73)	0.37								
Severe anemia	0.92 (0.28–3.02)	0.89								
Cerebral palsy	1.07 (0.60–1.88)	0.83								
Malaria	0.92 (0.39–2.19)	0.85								
Severe pneumonia	1.14 (0.73–1.79)	0.55								
***Daily clinical signs***										
Chest indrawing	2.89 (1.81–4.60)	<0.001	2.94 (1.91–4.52)	<0.001	0.15 (0.06–0.36)	<0.001	3.26 (2.02–5.25)	<0.001	3.44 (2.14–5.53)	<0.001
Convulsions	1.68 (0.71–3.98)	0.24								
Diarrhea	1.54 (1.03–2.32)	0.04	1.54 (1.04–2.27)	0.03	0.10 (0.05–0.17)	<0.001	1.83 (1.21–2.76)	0.00	1.82 (1.21–2.72)	0.00
Fever	1.59 (0.97–2.59)	0.06	1.52 (0.95–2.44)	0.08	0.09 (0.04–0.19)	<0.001	1.95 (1.21–3.14)	0.01	1.91 (1.19–3.09)	0.01
Hypothermia	1.11 (0.52–2.39)	0.79								
Not able to complete feeds	2.37 (1.54–3.65)	<0.001	2.50 (1.63–3.84)	<0.001	0.42 (0.32–0.55)	<0.001	3.58 (2.14–6.01)	<0.001	3.25 (2.01–5.27)	<0.001
Nutritional edema	1.73 (1.03–2.88)	0.04	1.66 (1.01–2.74)	0.04	0.22 (0.15–0.33)	<0.001	2.12 (1.31–3.43)	0.00	1.92 (1.21–3.06)	0.01
Reduced conciousness	6.89 (3.84–12.36)	<0.001	3.92 (1.88–8.15)	<0.001	0.39 (0.05–2.82)	0.35	5.46 (3.54–8.42)	<0.001	5.65 (3.66–8.73)	<0.001
Shock	1.35 (0.67–2.70)	0.40								
Symptomatic hypoglycemia	3.70 (1.73–7.88)	<0.001	4.18 (2.06–8.48)	<0.001	0.00 (0–Inf)	0.99	2.90 (1.3–6.47)	0.01	2.99 (1.32–6.75)	0.01
Vomitting	1.05 (0.65–1.70)	0.85								
Reduced consciousness: time^a^			1.15 (1.03–1.28)	0.01						
Observations (*n*)	6806		6852		6852		14381		14349	
Events (*n*)	124		124		637		124		124	
*P* of PH-test^b^ for mortality models	0.84		0.45		Not applicable		Not applicable		Not applicable	

Notes: *CWS* Clinical warning signs, *HR* Cause-specific hazard ratio, *PH* Proportional hazard, *HIV-* HIV-negative, *HIV+* HIV-positive. ^a^Linear function of time. ^b^Scaled Schoenfeld residuals test

Results of the competing risk discharge model (Table 2: Reduced Discharge Model) showed that all 7 CWS along with the a priori predictors were either negatively or not associated with hospital discharge. This means that the presence of any of these signs at any given time during hospitalization increased the daily hazard of dying (HR_death_>1) and decreased the daily hazard of being discharged (HR_discharge_<1). Therefore, although the cumulative incidence function cannot be estimated, it can still be anticipated that each of the selected CWS would exhibit a net positive association with risk of mortality (i.e., sub-distribution HR), after accounting for the competing risk effect of hospital discharge. For example, having chest indrawing at any given time of hospitalization was associated with an increased hazard of dying (HR_death_=2.9, *P*<0.001) and a decreased hazard of being discharged (HR_discharge_=0.2, *P*<0.001), which in turn indirectly increased the risk of later inpatient mortality, resulting in an overall stronger positive association between chest indrawing and risk of mortality. Manually setting the CWS to “not present” or to the discharge value in sensitivity analyses yielded higher HRs as compared to the extended Cox PH model. This implies that the HRs in the extended Cox PH model were not overestimated which suggests that competing risk does not here pose a problem.

Model diagnostics did not suggest evidence of overfitting and no significant interaction was found for the a priori defined clinically relevant interactions (data available upon request).

### Using CWS for daily mortality risk assessment

Predictive Model 2: Daily Score was built to evaluate the value of daily monitoring of the CWS identified above for risk assessment (Table 3). The C-index of Predictive Model 2: Daily Score was 0.81 (95% CI 0.77–0.86), which is the average prediction accuracy of using model-based day-specific risk scores to predict survival status of the respective score day. To estimate how much added value assessing CWS daily has, compared to only once upon admission, we also examined the prediction performance of the CWS upon admission. When using only the admission CWS to predict survival outcome, the C-index was 0.69 (95% CI 0.63–0.74) (Table 3). It is not surprising that the performance for these single-time scores were lower than for the daily scores, because the admission scores need to cover for a longer prediction time window (i.e., from admission to the end of study) than the daily scores (i.e., same day). As illustrated in Additional file 7: Figure S5, the performance of single-time scores decreased as the prediction time window increased.

**Table 3:** Predictive models based on identified clinical warning signs (CWS)

	Predictive Model 1: Admission Score		Predictive Model 2: Daily Score		Predictive Model 3: Daily Count (among all 8 identified CWS)		Predictive Model 4: Daily Count among Top 5 CWS	
	HR (95% CI)	*P*	HR (95% CI)	*P*	HR (95% CI)	*P*	HR (95% CI)	*P*
***CWS (in order of importance, as determined by decreasing HR in Predictive Model 2)***								
1 Reduced conciousness	3.6 (1.87–6.95)	<0.001	7.04 (4.25–11.67)	<0.001				
2 Symptomatic hypoglycemia	3.65 (1.66–8.06)	0.001	4.74 (2.47–9.11)	<0.001				
3 Chest indrawing	1.93 (1.26–2.96)	0.002	3.33 (2.18–5.09)	<0.001				
4 Not able to complete feeds	0.71 (0.49–1.05)	0.09	2.42 (1.58–3.7)	<0.001				
5 MUAC <10.5cm^a^	1.7 (1.18–2.45)	0.005	1.82 (1.25–2.66)	0.002				
6 Diarrhea	1.46 (1.03–2.08)	0.04	1.8 (1.23–2.62)	0.002				
7 Nutritional edema	1.59 (1.06–2.38)	0.02	1.62 (1.01–2.59)	0.047				
8 Fever	0.89 (0.57–1.4)	0.62	1.37 (0.88–2.15)	0.16				
***Counted number of CWS***^***b***^								
0					1.00 (ref)		1.00 (ref)	
1					4.67 (1.8–12.15)	0.002	3.44 (1.92–6.18)	<0.001
2					7.06 (2.67–18.68)	<0.001	10.68 (5.83–19.56)	<0.001
3					28.02 (10.84–72.46)	<0.001	46.51 (24.83–87.13)	<0.001
>3					100.38 (39.13–257.51)	<0.001	177.29 (81.27–386.79)	<0.001
Observations (*n*)	770		6852		6852		6852	
Events (*n*)	126		124		124		124	
C-index (95% CI)^c^	0.69 (0.63–0.74 )		0.81 (0.77–0.86)		0.79 (0.75–0.84)		0.79 (0.74–0.84)	

Notes: Data are estimation results from extended Cox proportional hazard models with (counted) CWS, event death and censored at discharge. *HR* cause-specific hazard ratio. ^a^As MUAC was only measured at admission, the counted number of the other CWS was increased by 1 on each hospitalization day if the child had a MUAC<10.5cm at admission. ^b^Counted from: 1 reduced consciousness, 2 symptomatic hypoglycemia, 3 chest indrawing, 4 not able to complete feeds, 5 MUAC<10.5cm, 6 diarrhea, 7 nutritional edema, and 8 fever. ^c^Bootstrapped with 1000 replications

### Counting CWS for daily mortality risk assessment

**Fig. 1:**
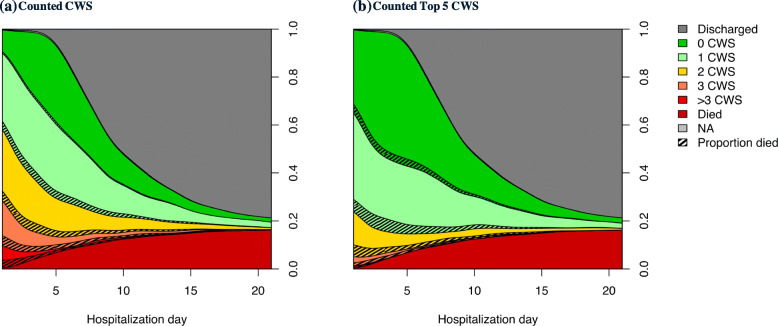
Dynamics in number of daily clinical warning signs (CWS) and survival outcome. Conditional density plot of the number of CWS and outcome (discharged versus died) among 780 SAM patients. The number of observed CWS were counted from **a** all 8 identified CWS (reduced consciousness, symptomatic hypoglycemia, chest indrawing, not able to complete feeds, MUAC<10.5cm, diarrhea, nutritional edema, and fever) and **b** the top 5 CWS (reduced consciousness, symptomatic hypoglycemia, chest indrawing, not able to complete feeds, and MUAC<10.5cm). The hatch area within each CWS count category indicates the proportion of patients who eventually died during hospitalization

Figure 1 shows the dynamics of the number of counted CWS during hospitalization and the trajectory towards dying among the counted number of daily CWS, as illustrated by the proportion of subjects who eventually died during hospitalization in each category. This figure also shows how the number of CWS changed over time. For example, Fig. 1a shows that the proportion of children with more than 3 CWS (category in red color) decreased rapidly during hospitalization, since half of the children in this category died (shaded area) and exited the study population. In addition, there is an expansion of children with 1 CWS (category in light green) around day 4 resulting from previous CWS being resolved with treatment. Although there is an overall reducing trend in the number of CWS during hospitalization, a small proportion of children showed clinical deterioration with increasing CWS. Namely, 16% of the children had an increase of 2 or more in CWS after admission. More descriptive statistics on the changes can be found in Additional file 8: Table S3.

Table 3 shows the association between the counted number of CWS and mortality (Predictive Model 3 and Predictive Model 4), where 7 identified daily CWS were counted together with MUAC<10.5cm at admission, and their importance was ranked in Predictive Model 2 by decreasing HR. Counting from the top 5 CWS (1 reduced consciousness, 2 symptomatic hypoglycemia, 3 chest indrawing, 4 not able to complete feeds, and 5 MUAC <10.5cm), the daily hazard of dying during hospitalization among patients with 1, 2, 3, and more than 3 counted signs was 3.4 (95% CI 1.9–6.2; *P<*0.001), 10.7 (95% CI 5.8–19.6; *P*<0.001), 46.5 (95% CI 24.8–87.1; *P*<0.001), and 177.3 (95% CI 81.3–386.8; *P*<0.001) times higher, respectively, than among children with none of these 5 signs. Comparing the counting tool with the observed data showed that all children who died had at least 1 of the 8 identified CWS; only 10 death cases had none of the top 5 CWS, but those did have either edema and/or diarrhea.

Performance as measured by C-index was similar between counting all 8 identified CWS and the top 5 CWS (Table 3). Estimating Predictive Model 4 among children with the age range restricted to 6 to 59 months (*n*=738) in sensitivity analysis yielded similar prediction performance (C-index of 0.79; bootstrapped 95% CI 0.75–0.84). In further sensitivity analysis, we estimated Predictive Model 4 on the Kenyan and Malawian subsamples, respectively, which showed a slightly higher prediction performance for Kenya (C-index Kenya: 0.83 versus C-index Malawi 0.78), but the difference was not significant (bootstrapped 95% CI Kenya: 0.77–0.90 and bootstrapped 95% CI Malawi: 0.72–0.85).

**Fig. 2:**
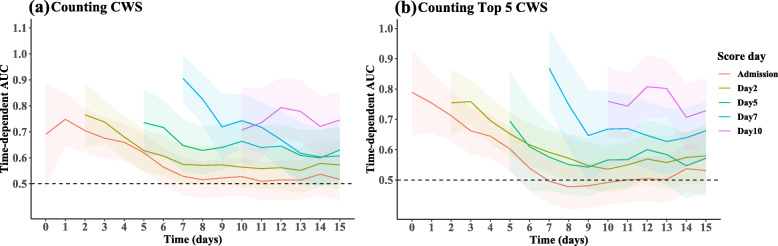
Performance of counting scores evaluated on selected landmarking days over time. **a** Time-dependent AUC of using the number of CWS (counted among reduced consciousness, symptomatic hypoglycemia, chest indrawing, not able to complete feeds, MUAC<10.5cm, diarrhea, nutritional edema, and fever) as risk scores assessed on a specific day (admission, days 2, 5, 7, 10) to predict survival outcome for the subsequent days (including the score day) up to 15 days since admission. **b** Time-dependent AUC of using the number of the top 5 CWS (counted among reduced consciousness, symptomatic hypoglycemia, chest indrawing, not able to complete feeds, and MUAC<10.5cm) as risk scores assessed a specific day (admission, days 2, 5, 7, and 10) to predict survival outcome for the subsequent days (including the score day) up to 15 days since admission. AUC=0.5 implies performance is no better than random chance

Figure 2 shows the time-dependent prediction accuracy (t-AUC) for counting CWS on set score days (admission, days 2, 5, 7, and, 10 of hospitalization) to make predictions for the days following the score day. As previously noted, performance for scores measured at a single time decreases over time, substantiating the importance of continuous assessment to maintain the prognostic accuracy. For instance, the CWS counts assessed on day 2 can predict mortality occurring by the end of day 2 with an AUC of 0.82, but to predict mortality during the following 3 days (i.e., by the end of day 5), the AUC dropped to 0.63. In general, prediction performance remains above an AUC of 0.7 within 48 h of assessment. Counting just the top 5 CWS attained similar accuracy as counting all 8 identified CWS at each of the score days. In addition, counting the top 5 CWS attained similar accuracy as model-based scores (Additional file 9: Figure S6).

## Discussion

To our knowledge, this is the first study that evaluated the use of daily CWS to predict inpatient mortality among children with SAM. We identified 7 daily CWS that are associated with increased risk of mortality, including symptomatic hypoglycemia, reduced consciousness, chest indrawing, not being able to complete feeds, nutritional edema, diarrhoea, and fever. When using these daily CWS together with MUAC<10.5cm at admission to assess mortality risk, the C-index was 0.81 (95% CI 0.77–0.86). This prediction accuracy suggests that sick children with complicated SAM who are at high risk of dying can be reasonably captured by the presence of these CWS. As expected, this prediction performance was higher than when using only the admission score of these CWS to predict survival outcome, underscoring the importance of systematically assessing CWS daily to update dynamics in patient status during hospitalization. Counting CWS among the top 5 signs (reduced consciousness, symptomatic hypoglycemia, chest indrawing, not able to complete foods, and MUAC<10.5cm) provided a simpler tool for assessing patient status, which has reasonable prognostic accuracy for both same-day prediction (C-index of 0.79; 95% CI 0.74–0.84), and a 48-h prediction (average t-AUC>0.7). Having 1 or 2 of these 5 CWS on any day during hospitalization was associated with a 3- or 11-fold increase in hazard of mortality compared with no signs, respectively.

The classic focus for mortality prediction using admission data makes sense as, according to the literature, many patients with SAM die in the first 48 h after being admitted to a hospital (SAM [30], while another paper on mortality in non-SAM reported that 87% of all mortality in that study occurred within 24 h of admission [31]). In the present study, a relatively small proportion of mortality (14%) happened in the first 48 h showing the relevance of using the daily CWS for mortality prediction. Moreover, the clinical course of a patient is expected to change daily, which would be missed using admission data only. This is confirmed by a previous study done in Kenya where it was shown that wasting and kwashiorkor were not associated with early mortality but were strongly associated with late mortality [32]. Our data are also in line with another study done in Kenya, in which 33% of deaths happened in the first 48 h after admission, the rest later into admission, and after 21 days of admission no significant mortality occurred [12]. Late mortality among children with SAM calls for continued, daily monitoring of the clinical status and subsequent risk prediction during the entire admission. The decrease in performance that we saw with increasing prediction time window substantiates the importance of structured monitoring of clinical signs on a daily basis.

The a priori risk factor that was found to increase the risk of mortality in children with SAM was MUAC, and this confirms what is already known [3]. Our data confirms several CWS at admission that are associated with mortality in children with SAM. Talbert et al. found diarrhea to be strongly associated with mortality [8]. Reduced consciousness and hypoglycemia were also found linked to death by Maitland et al. [12]. Girum et al. found hypoglycemia and fever at admission to be associated with mortality in children with SAM [13]. These and many other studies on risk prediction in children with SAM have focussed on the use of CWS upon admission (only). In addition to the use of CWS in children with SAM, risk prediction at admission has also been done among children without severe malnutrition. Low anthropometry and reduced consciousness were mentioned as risk factors by O’Reilly in non-malnourished children with diarrhea [33], while low anthropometry, reduced consciousness, respiratory distress, and fever were found to be risk factors in a study in children with severe pneumonia [34]. George et al. identified a subset of 8 parameters, among which respiratory distress and altered consciousness, as predictors (at admission) of inpatient mortality in non-malnourished children [35]. A recent paper on prediction modeling of neonatal mortality in low- and middle-income countries (LMICs) confirmed that it is possible to predict in-hospital mortality in neonates as well [36].

A recent systematic review by Ogero et al. identified 21 models predicting in-hospital pediatric mortality in LMICs [37]. Of note, all these 21 models used clinical signs on admission, while none used daily clinical data to predict outcomes later during hospitalization. These models have gained limited utility to date. Most models had several important methodological concerns, such as a priori selection of predictors and ignoring censoring with the use of logistic models. Some models require monitoring of vital signs or laboratory measurements, which pose difficulties to implementation [37]. Therefore, the present study is unique in its inclusion of daily clinical parameters (not selected beforehand) capturing disease dynamics throughout hospitalization in predicting in-patient mortality.

Earlier attempt to provide healthcare workers in LMICs with a simple bedside score are not new [12, 32] but were constructed for use on admission only and have not been taken up in practice. Results from this study could address this gap, since counting CWS from the top 5 signs (reduced consciousness, symptomatic hypoglycemia, chest indrawing, not able to complete feds, and MUAC<10.5cm at admission) provides a simple tool with adequate prognostic performance. A patient with SAM having any of these top 5 CWS should be more frequently reviewed clinically and medically investigated further, and treatment should be adjusted accordingly. Future planned trials will show whether using this simple CWS counting tool will lead to improved care, more appropriate use of resources, and improved outcome for vulnerable malnourished children. We propose that recommendations in the current (WHO) clinical management guidelines on detecting failure-to-improve or clinical deterioration should be reconsidered, guided by focusing on the 5 key CWS identified in this study for the standard daily practice for these vulnerable children.

Site influences mortality, with risk being higher in Malawi, as compared to both Kenyan sites. Additional file 10: Table S4 compares patient characteristics upon admission between Malawi and Kenya, suggesting that, while children in Malawi presented with less clinical signs, they generally had worse nutritional status (i.e., more edema, lower non-edematous MUAC, more severely wasting children, and lower HAZ, but a higher WHZ), and higher HIV prevalence or reactivity. However, the sensitivity analysis comparing the mortality prediction performance for Kenya versus Malawi showed similar prediction performance in both countries, justifying application of the prediction tool to both sites.

HIV+/exposed was not associated with mortality in our study population, after adjusting in the explanatory models for site, MUAC, and signs of illness severity. Five percent of children had an unknown HIV status, but these were not missing at random as several missing tests (33%) were linked to either early death cases (i.e., within 2 days of admission), carers of participants potentially refusing further testing because of their known HIV status, or simple refusal of the test. Multiple imputation strategies were explored but yielded poor results and thus not further considered (data available upon request). Thus, we have chosen to exclude HIV status from the four predictive models, considering both the missingness pattern, and the fact that HIV+/exposed was not significant in the explanatory models.

Although shock is commonly associated with mortality in other settings [31, 38, 39], it was not found to be associated with time-to-death in the present study. Shock was recorded daily as the composite of fast and weak pulse, cold hands, and capillary refill time more than 3 s in the last 24h (Additional file 1: Table S1). Being a composite sign, shock may have greater heterogeneity than other signs. Shock was also much less common than other signs detected during hospitalization, as showed in Additional file 5: Figure S3. These may explain why shock was not selected in the multivariable survival analysis when adjusted for covariates.

In our study, SAM was identified based on WHO criteria which uses both MUAC and WHZ. In the analyses of warning signs associated with mortality, we chose to focus on MUAC for pragmatic reasons. Unlike WHZ, MUAC is more practical (i.e., does not require a weight scale and height board, nor complex calculations) and produces more accurate measurements in very sick children as it is less affected by hydration status [20]. While comparing anthropometric measures was not the focus of our study, we did evaluate WHZ as a baseline predictor in sensitivity analyses. In line with some other studies [40–43], in this study, MUAC outperformed WHZ in identifying children at high risk of death. For example, when both measures were included in the explanatory Full Mortality Model, MUAC but not WHZ was retained as significant predictor using backward feature selection. Additionally, prediction performance using MUAC<10.5 was consistent across different age groups, substantiating the inclusion of MUAC as a warning sign in the development of a practical monitoring tool.

Although the present study is the first to analyze daily CWS, we acknowledge that this is a secondary analysis of clinical trial data and this is not without limitations. First, CWS were monitored every 24 h during daily clinical ward rounds, which may not be frequent enough to capture all clinical variation. Secondly, caution is needed when generalizing the present findings to other populations. The results of this study will be validated in a cohort of Asian children with complicated SAM, where HIV prevalence is much lower [44]. Lastly, post-discharge follow-up was not performed in this trial and as a result we do not know how well the CWS predict post-discharge mortality, whereas many deaths in children with SAM occur shortly after discharge [45–47].

## Conclusions

Monitoring CWS on a daily basis improved the accuracy of predicting mortality in children with SAM, compared to using admission predictors only. Additionally, having 2 of 5 key CWS on any day during hospitalization was associated with an 11-fold increase in the hazard of death on that day. These results underscore that counting CWS could serve as an easily applicable tool for identification of changes in risk over time [48], similar to pediatric early warning signs (PEWS) used in high-income settings [49]. This is highly relevant in low-resource settings where number of health care workers per patient is low, workload is therefore high, and appropriate resource allocation may impact child survival. With currently unacceptably high inpatient mortality despite treatment reported from hospital settings, results from this study (1) may help standardizing daily assessment for patient vulnerability, (2) can inform a standardized daily assessment (ward rounds) of progress or failure to respond to treatment, and (3) may help in improving the current WHO management guidelines.

## Supplementary Information

**Additional file 1: Table S1.** Definitions of daily clinical signs.

**Additional file 2: Figure S1.** Illustration C-index computation for time-static and time-updated predictions based on survival status of hypothetical subjects. (a) Concordance of Time-static Prediction. Illustration of concordance computation in the scenario where risk assessment is made once at a single time point (e.g., admission) for ultimate survival outcome prediction. (b) Concordance of Time-updated Prediction. Illustration of concordance computation in the scenario where risk assessment is repeated every day and the updated risk score is used for survival prediction. Survival information pertaining to the five hypothetical subjects is colored in blue; filled triangles denote occurrence of death (event), empty triangles denote occurrence of discharge (censoring), empty circles denote subjects remaining at risk at a given time point. Solid black arrows indicate valid pairs of risk score comparisons contributing to concordance computation.

**Additional file 3: Figure S2.** Kaplan-Meier survival curve and risk table of the study population before restricting data to 21 days.

**Additional file 4: Table S2.** Missing data on daily clinical warning signs.

**Additional file 5: Figure S3.** Dynamics in the individual clinical signs and survival outcome (conditional density plots).

**Additional file 6: Figure S4.** Scaled Schoenfeld residuals plot of reduced consciousness against the transformed time. Solid black line denotes the smoothing spline fit to residuals of the coefficient for variable reduced consciousness (beta(t)), with the dashed lines indicating a ±2 standard error band. The solid black line systematic departures from the horizontal green line, suggesting non-proportional hazards (i.e., time-dependent effect) of reduced consciousness. Red line is the estimated time-dependent coefficient of reduced consciousness (β(t)= 1.37+ 0.14*t) for the Reduced Mortality Model.

**Additional file 7: Figure S5.** Performance of model-based scores evaluated on selected landmarking days over time. Time-dependent AUC of using risk scores (calculated from Predictive Model 2: Daily Score) assessed on a specific day (admission, day 2, 5, 7, 10) to predict survival outcome for the subsequent days (including the score day) up to 15 days since admission. AUC=0.5 implies performance is no better than random chance.

**Additional file 8: Table S3.** Proportion of children with changes in number of CWS between two consecutive hospitalization days.

**Additional file 9: Figure S6.** Performance of day-specific risk scores of different predictive models in predicting survival outcome in the subsequent 2 days. AUC of risk scores at different score days (admission, day 1, day 2, …, day 15) calculated from the three predictive models in discriminating deaths for the subsequent 2 days (including the score day). AUC=0.5 implies performance is no better than random chance.

**Additional file 10: Table S4.** Patient characteristics at admission, by country.

## Data Availability

Data files of the clinical trial are available through Berkley, J. A., Bandsma, R. H. J., and Ngari, M. M. Modified F75 formula for stabilization among hospitalized children with severe acute malnutrition: double-blind, randomized controlled trial. Harvard Dataverse 10.7910/DVN/N4RISX (2019). All other materials are available from the corresponding author on reasonable request.

## Abbreviations

95% CI: 95% Confidence intervals
CHAIN: Childhood Acute Illness Network
C-index: Concordance index
CWS: Clinical warning signs
DFBETA: Difference in the β coefficient
ETAT: Emergency triage assessment and treatment
HIV+/exposed: HIV-positive or positive antibody reactivity
HR: Hazard ratio
LMIC: Low- and middle-income countries
MUAC: Mid-upper arm circumference
PEWS: Pediatric early warning signs
PH: Proportional hazards
SAM: Severe acute malnutrition
SD: Standard deviation
t-AUC: Time-dependent area under the receiver operating characteristic curve
VIF: Variance inflation factor
WHO: World Health Organization
WHZ: Weight-for-height *Z* score
