# Supplementary material for: Predicting the risk of mortality during hospitalization in sick severely malnourished children using daily evaluation of key clinical warning signs
Source: BMC Med. 2021 Sep 20;19:222. doi: 10.1186/s12916-021-02074-6 (PMC8451091; doi:10.1186/s12916-021-02074-6)
Supplement: Supplementary file 1 — Additional file 1: Table S1. Definitions of daily clinical signs. [file 12916_2021_2074_MOESM1_ESM.docx]

| **Additional File 1: Table S1. Definitions of daily clinical signs** | |
| --- | --- |
| ***Daily clinical signs*** |  |
| Diarrhea | ≥3 loose stools in the last 24h |
| Fever | Temperature >38.5^o^C in the last 24h |
| Vomiting | Vomiting in the last 24h |
| Chest indrawing | Lower chest wall indrawing in the last 24h |
| Hypothermia | Temperature <36.5^o^C Celsius in the last 24h |
| Convulsions | Convulsions in the last 24h |
| Shock | Fast and weak pulse, cold hands, and capillary refill time >3 seconds in the last 24h |
| Reduced consciousness | Lowest AVPU score being P or U in the last 24h |
| Symptomatic hypoglycemia | <3 mmol/l in the last 24h; glucose measured systematically at admission and subsequently only when clinicians suspected hypoglycemia |
| Nutritional edema | Presence of edema in the last 24h |
| Not able to complete feeds | Not able to complete foods in the last 24h |
