# Supplementary material for: Predicting the risk of mortality during hospitalization in sick severely malnourished children using daily evaluation of key clinical warning signs
Source: BMC Med. 2021 Sep 20;19:222. doi: 10.1186/s12916-021-02074-6 (PMC8451091; doi:10.1186/s12916-021-02074-6)
Supplement: Supplementary file 2 — Additional file 2: Figure S1. Illustration C-index computation for time-static and time-updated predictions based on survival status of hypothetical subjects. (a) Concordance of Time-static Prediction. Illustration of concordance computation in the scenario where risk assessment is made once at a single time point (e.g., admission) for ultimate survival outcome prediction. (b) Concordance of Time-updated Prediction. Illustration of concordance computation in the scenario where risk assessment is repeated every day and the updated risk score is used for survival prediction. Survival information pertaining to the five hypothetical subjects is colored in blue; filled triangles denote occurrence of death (event), empty triangles denote occurrence of discharge (censoring), empty circles denote subjects remaining at risk at a given time point. Solid black arrows indicate valid pairs of risk score comparisons contributing to concordance computation. [file 12916_2021_2074_MOESM2_ESM.pdf]

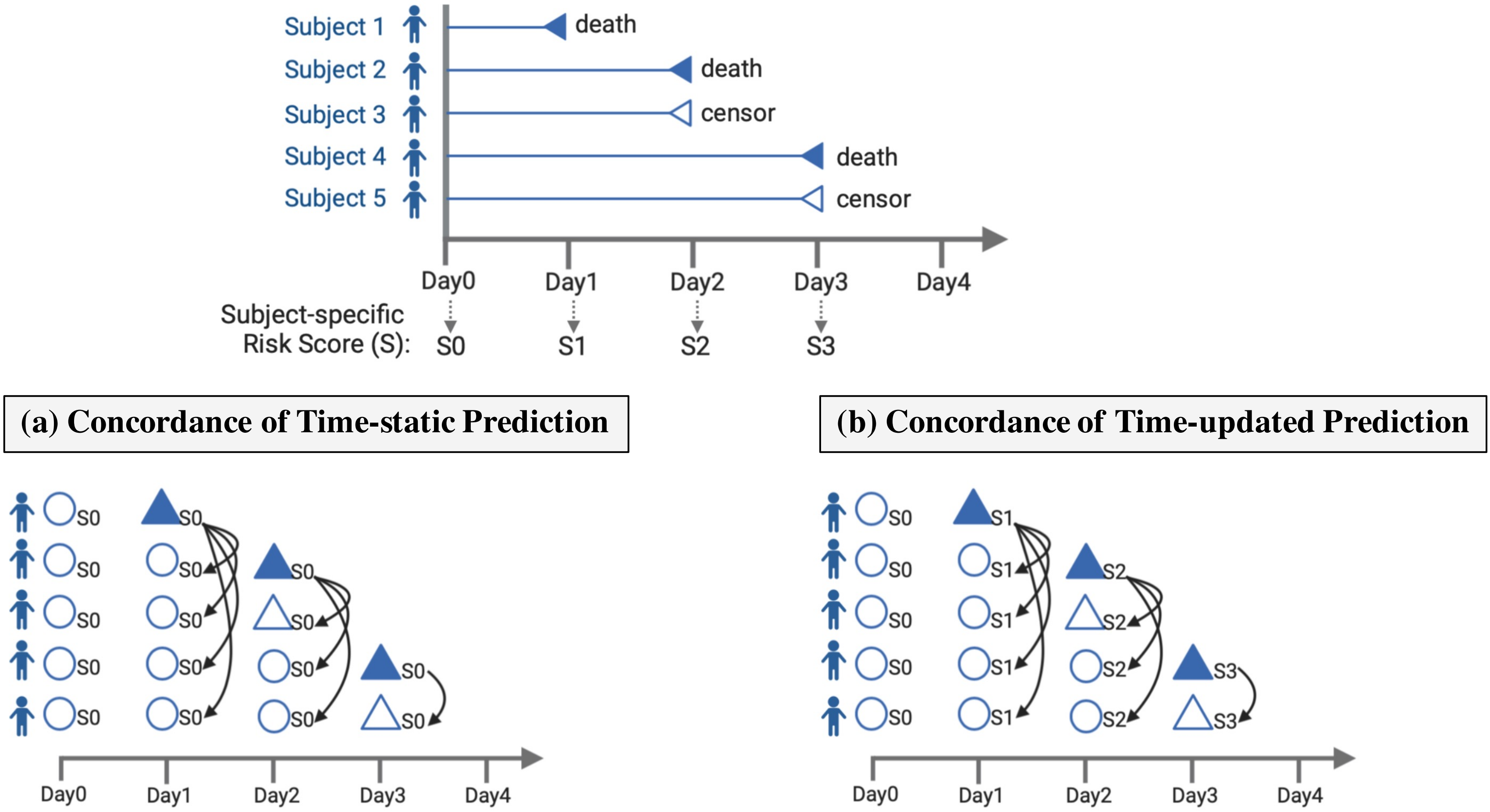

A valid pair of concordant comparison: ↷

Concordant pair: Risk score of subject died > Risk score of subject alive

Discordant pair: Risk score of subject died ≤ Risk score of subject alive

Concordance index = Total Concordant pairs / Total Discordant pairs
