# Supplementary figures and images for: Predicting the risk of mortality during hospitalization in sick severely malnourished children using daily evaluation of key clinical warning signs
Source: BMC Med. 2021 Sep 20;19:222. doi: 10.1186/s12916-021-02074-6 (PMC8451091; doi:10.1186/s12916-021-02074-6)

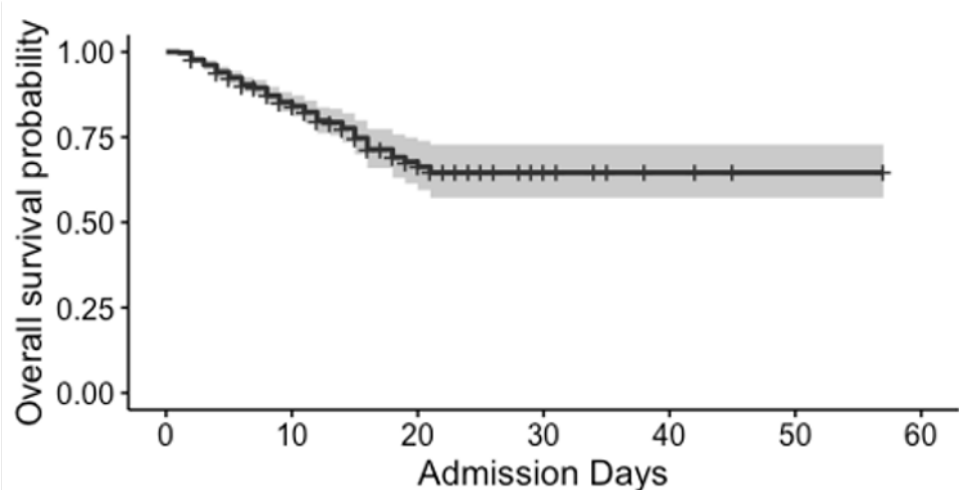

Number at risk

|     |     |    |    |   |   |   |
|-----|-----|----|----|---|---|---|
| 780 | 286 | 46 | 12 | 3 | 1 | 0 |
|-----|-----|----|----|---|---|---|

Cumulative number of events

|   |    |     |     |     |     |     |
|---|----|-----|-----|-----|-----|-----|
| 0 | 99 | 126 | 127 | 127 | 127 | 127 |
|---|----|-----|-----|-----|-----|-----|

Cumulative number of censoring

|   |     |     |     |     |     |     |
|---|-----|-----|-----|-----|-----|-----|
| 0 | 443 | 616 | 642 | 650 | 652 | 653 |
|---|-----|-----|-----|-----|-----|-----|

Admission Days

Supplement: Supplementary file 3 — Additional file 3: Figure S2. Kaplan-Meier survival curve and risk table of the study population before restricting data to 21 days. [file 12916_2021_2074_MOESM3_ESM.pdf]

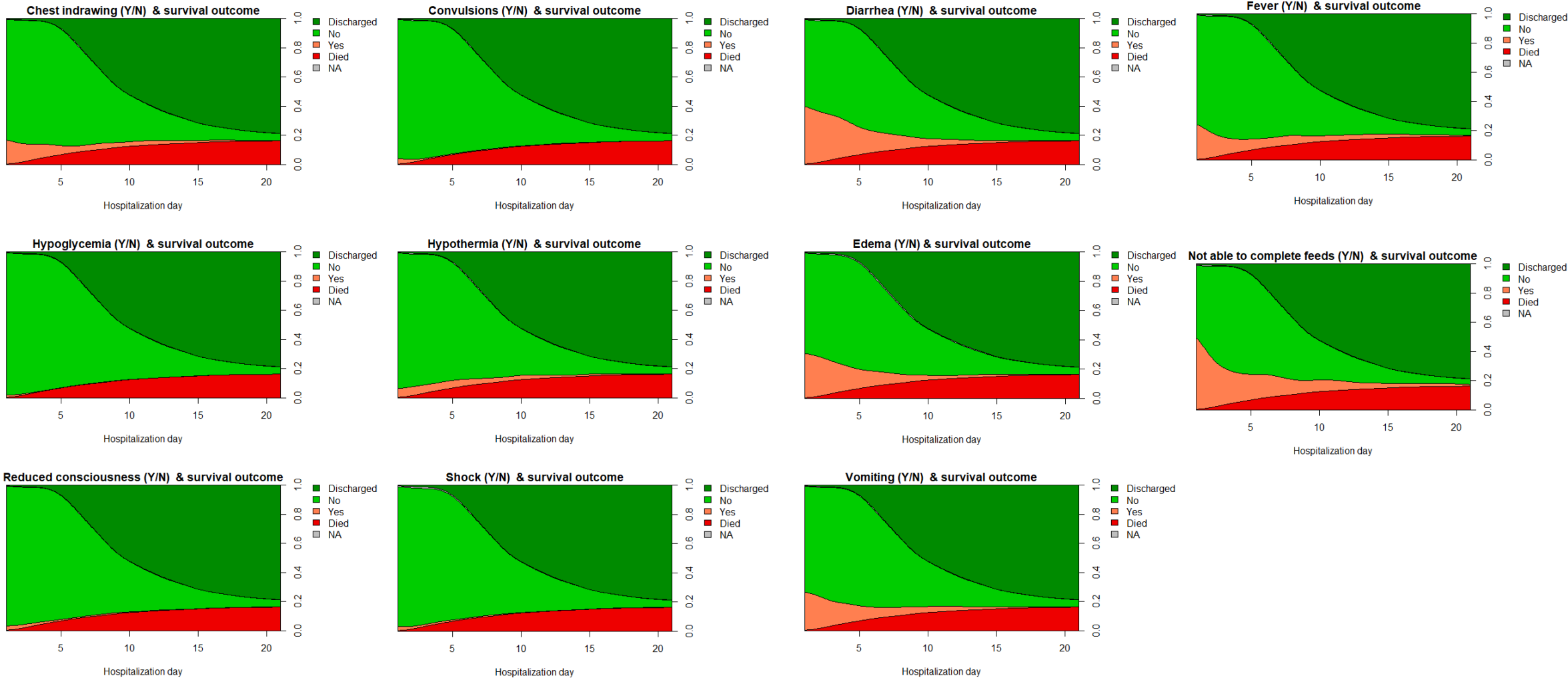

Supplement: Supplementary file 5 — Additional file 5: Figure S3. Dynamics in the individual clinical signs and survival outcome (conditional density plots). [file 12916_2021_2074_MOESM5_ESM.pdf]

Beta(t) for Reduced Consciousness

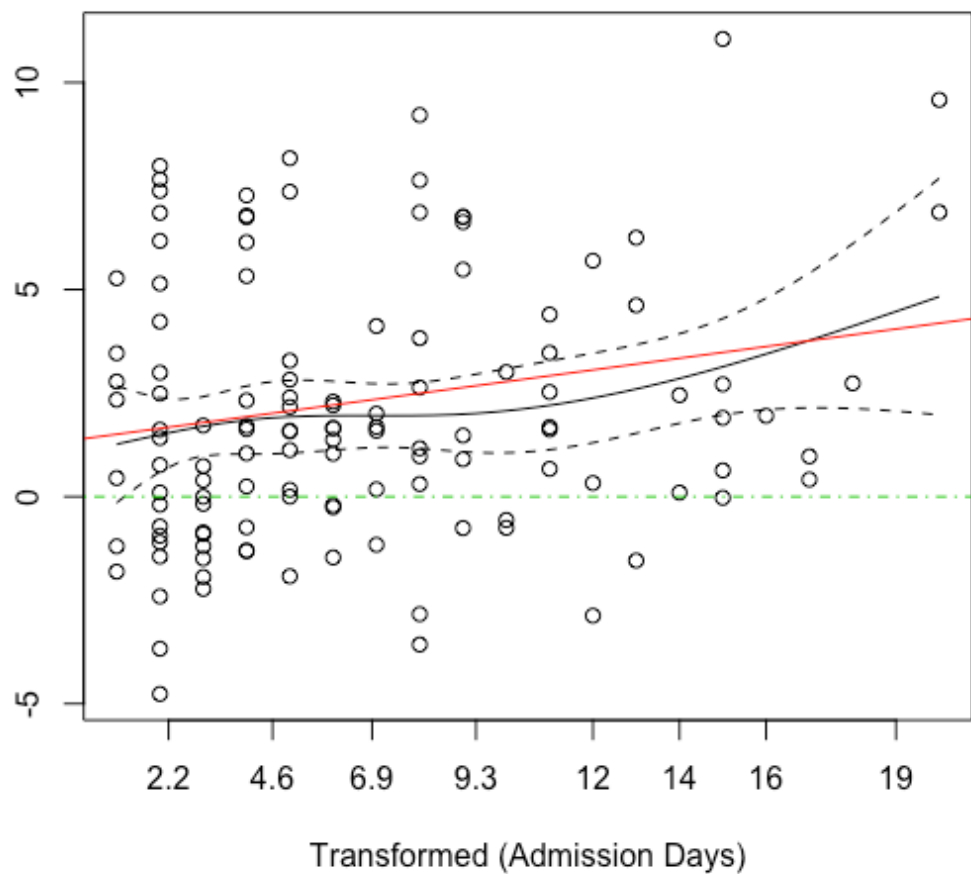

Supplement: Supplementary file 6 — Additional file 6: Figure S4. Scaled Schoenfeld residuals plot of reduced consciousness against the transformed time. Solid black line denotes the smoothing spline fit to residuals of the coefficient for variable reduced consciousness (beta(t)), with the dashed lines indicating a ±2 standard error band. The solid black line systematic departures from the horizontal green line, suggesting non-proportional hazards (i.e., time-dependent effect) of reduced consciousness. Red line is the estimated time-dependent coefficient of reduced consciousness (β(t)= 1.37+ 0.14*t) for the Reduced Mortality Model. [file 12916_2021_2074_MOESM6_ESM.pdf]

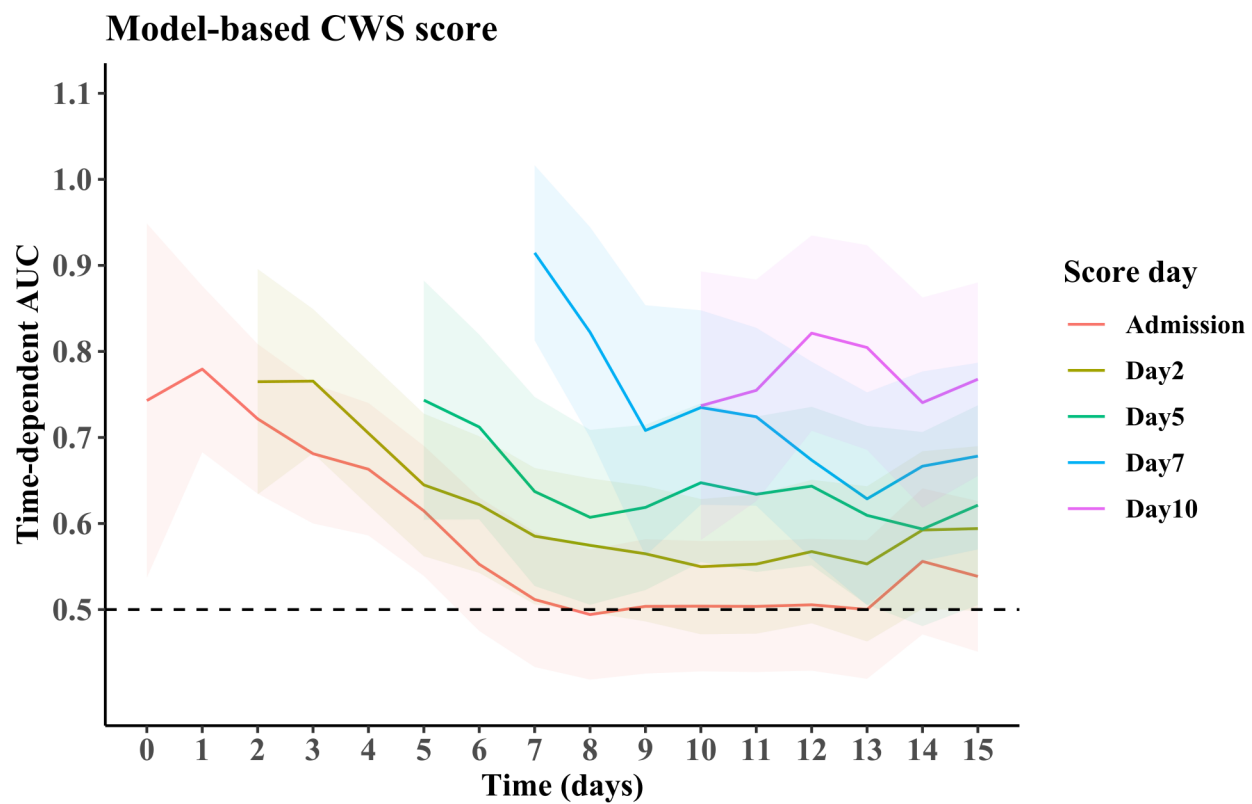

Supplement: Supplementary file 7 — Additional file 7: Figure S5. Performance of model-based scores evaluated on selected landmarking days over time. Time-dependent AUC of using risk scores (calculated from Predictive Model 2: Daily Score) assessed on a specific day (admission, day 2, 5, 7, 10) to predict survival outcome for the subsequent days (including the score day) up to 15 days since admission. AUC=0.5 implies performance is no better than random chance. [file 12916_2021_2074_MOESM7_ESM.pdf]

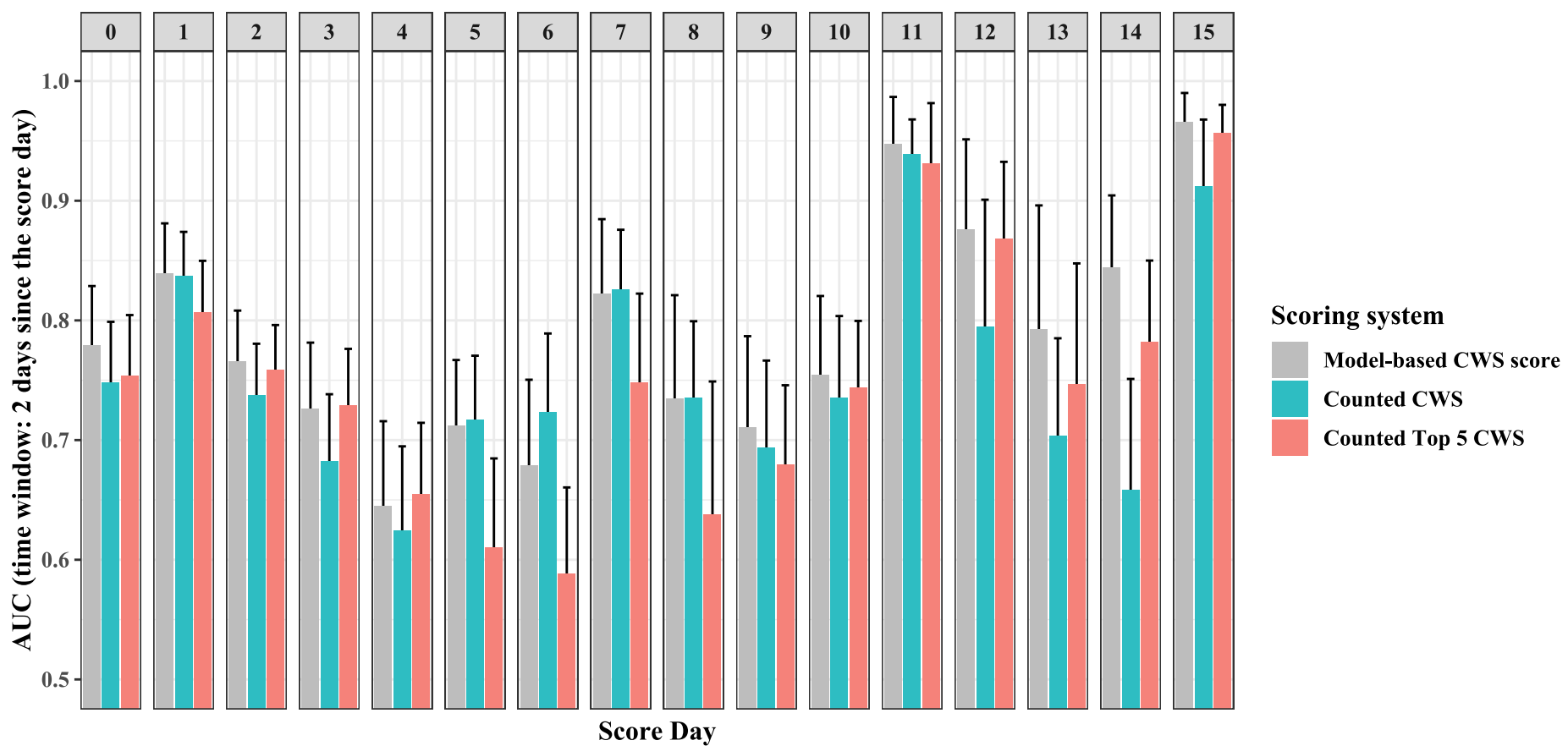

Supplement: Supplementary file 9 — Additional file 9: Figure S6. Performance of day-specific risk scores of different predictive models in predicting survival outcome in the subsequent 2 days. AUC of risk scores at different score days (admission, day 1, day 2, …, day 15) calculated from the three predictive models in discriminating deaths for the subsequent 2 days (including the score day). AUC=0.5 implies performance is no better than random chance. [file 12916_2021_2074_MOESM9_ESM.pdf]
