# Supplementary material for: Predicting the risk of mortality during hospitalization in sick severely malnourished children using daily evaluation of key clinical warning signs
Source: BMC Med. 2021 Sep 20;19:222. doi: 10.1186/s12916-021-02074-6 (PMC8451091; doi:10.1186/s12916-021-02074-6)
Supplement: Supplementary file 4 — Additional file 4: Table S2. Missing data on daily clinical warning signs. [file 12916_2021_2074_MOESM4_ESM.docx]

| **Additional File 4: Table S2. Missing data on daily clinical warning signs** | |
| --- | --- |
|  | **Total observations** |
|  | (*n*=7025) |
| ***Daily clinical warning signs*** |  |
| Diarrhea, n (%) | 88 (1.2) |
| Fever, n (%) | 98 (1.4) |
| Vomiting, n (%) | 90 (1.3) |
| Chest indrawing, n (%) | 83 (1.2) |
| Hypothermia, n (%) | 98 (1.4) |
| Convulsions, n (%) | 85 (1.2) |
| Shock, n (%) | 107 (1.5) |
| Reduced consciousness, n (%) | 82 (1.2) |
| Symptomatic hypoglycemia, n (%) | 86 (1.2) |
| Nutritional edema, n (%) | 123 (1.8) |
| Not able to complete feeds, n (%) | 95 (1.4) |
| Notes: data are number (%) of missing observations out of all observations. | |
