# Supplementary material for: Predicting the risk of mortality during hospitalization in sick severely malnourished children using daily evaluation of key clinical warning signs
Source: BMC Med. 2021 Sep 20;19:222. doi: 10.1186/s12916-021-02074-6 (PMC8451091; doi:10.1186/s12916-021-02074-6)
Supplement: Supplementary file 8 — Additional file 8: Table S3. Proportion of children with changes in number of CWS between two consecutive hospitalization days. [file 12916_2021_2074_MOESM8_ESM.docx]

| **Additional File 8: Table S3. Proportion of children with changes in number of CWS between two consecutive hospitalization days** | | | |
| --- | --- | --- | --- |
| Maximum increase^a^ | | Maximum decrease^b^ |  |
| No change | 0.37 | No change | 0.09 |
| Increase 1 sign | 0.46 | Decrease 1 sign | 0.52 |
| Increase 2 signs | 0.13 | Decrease 2 signs | 0.33 |
| Increase ≥3 signs | 0.03 | Decrease ≥3 signs | 0.06 |
| ^a^For all children with at least 2 hospitalization days (N=768), the maximum increase was calculated as the maximum observed increase in the number of CWS between 2 consecutive days using data of their whole hospitalization period. | | | |
| ^b^For all children with at least 2 hospitalization days (N=768), the maximum decrease was calculated as the maximum observed decrease in the number of CWS between 2 consecutive days using data of their whole hospitalization period. | | | |
