# Supplementary material for: Predicting the risk of mortality during hospitalization in sick severely malnourished children using daily evaluation of key clinical warning signs
Source: BMC Med. 2021 Sep 20;19:222. doi: 10.1186/s12916-021-02074-6 (PMC8451091; doi:10.1186/s12916-021-02074-6)
Supplement: Supplementary file 10 — Additional file 10: Table S4. Patient characteristics at admission, by country. [file 12916_2021_2074_MOESM10_ESM.docx]

| **Additional File 10: Table S4. Patient characteristics at admission, by country** | | | |
| --- | --- | --- | --- |
|  | **Malawi (1 site)** | **Kenya (2 sites)** |  |
|  | (*n*=311) | (*n*=469) | *P* |
| *Study related characteristics* |  |  |  |
| Mortality, n (%) | 64 (20.6) | 63 (13.4) | 0.008 |
| Hospital stay (days), median (IQR) | 6.0 (5.0, 8.0) | 9.0 (7.0, 13.0) | <0.001 |
| Treatment arm of trial, n (%) | 155 (49.8) | 235 (50.1) | 0.94 |
| *Demographic and anthropometric characteristics* |  |  |  |
| Age in months, median (IQR) | 21.6 (14.6, 31.5) | 14.1 (9.5, 22.5) | <0.001 |
| Age 6-59 months, n (%) | 293 (94.2) | 445 (94.9) | 0.69 |
| Male, n (%) | 164 (52.7) | 256 (54.6) | 0.61 |
| MUAC in cm (non-edematous), median (IQR) | *n*=140; 10.9 (10, 11.3) | *n*=391; 11 (10.5, 11.6) | 0.02 |
| MUAC in cm, median (IQR) | 11.3 (10.4-12.5) | 11 (10.5-11.7) | <0.001 |
| MUAC<10.5cm, n (%) | 79 (25.4) | 113 (24.1) | <0.001 |
| HAZ, median (IQR) | -3.3 (-4.4, -2.3) | *n*=465; -2.6 (-4.2, -1.5) | <0.001 |
| WAZ, median (IQR) | -4.0 (-4.9, -2.7) | *n*=467; -3.9 (-4.9, -3.2) | 0.61 |
| WHZ, median (IQR) | *n*=296; -3.2 (-4.2, -1.6) | *n*=441; -3.59 (-4.2, -3.0) | 0.001 |
| *Comorbidities (observed upon admission only)* |  |  |  |
| HIV status, n (%) |  |  |  |
| HIV- | 218 (70.1) | 353 (75.3) |  |
| HIV+/exposed | 86 (27.7) | 83 (17.7) |  |
| Refused testing/died before testing | 7 (2.3) | 33 (7.0) | <0.001 |
| Cerebral palsy, n (%) | 50 (16.8) | 66 (14.1) | 0.44 |
| Severe pneumonia, n (%) | 40 (12.9) | 153 (32.6) | <0.001 |
| Severe anemia, n (%) | 2 (0.6) | 24 (5.1) | 0.001 |
| Malaria, n (%) | 32 (10.3) | 31 (6.6) | 0.07 |
| ***Prevalence at admission of (daily observed) clinical signs*** |  |  |  |
| Chest indrawing, n (%) | 17 (5.5) | 127 (27.1) | <0.001 |
| Convulsions, n (%) | 5 (1.6) | 32 (6.8) | 0.001 |
| Diarrhea, n (%) | 114 (36.7) | 214 (45.6) | 0.01 |
| Fever, n (%) | 16 (5.1) | 200 (42.6) | <0.001 |
| Symptomatic hypoglycemia, n (%) | 4 (1.3) | 9 (1.9) | 0.50 |
| Hypothermia, n (%) | 7 (2.3) | 36 (7.7) | 0.001 |
| Nutritional edema, n/total non-missing (%) | 171 (55.0) | 75/466 (16.1) | <0.001 |
| Not able to complete feeds, n/total non-missing (%) | 76 (24.4) | 374/462 (81.0) | <0.001 |
| Reduced consciousness, n (%) | 3 (1.0) | 21 (4.5) | 0.005 |
| Shock, n (%) | 4 (1.3) | 21 (4.5) | 0.01 |
| Vomiting, n (%) | 73 (23.5) | 142 (30.3) | 0.04 |
| Notes: data are median (IQR) or number (%) of SAM patients, by site country (Malawi vs. Kenya). HAZ = height-for-age z-score. WAZ = weight-for-age z-score. WHZ = weight-for-height z-score. HIV- = HIV negative. HIV+/exposed = HIV positive or positive antibody reactivity. | | | |
